# One-Dimensional Nickel Molybdate Nanostructures with Enhanced Supercapacitor Performance

**DOI:** 10.3390/polym15234538

**Published:** 2023-11-26

**Authors:** Baodong Sun, Shaomin Wang, Mingyi Zhang

**Affiliations:** 1 College of Teacher Education, Harbin Normal University, Harbin 150025, China; 2 Key Laboratory for Photonic and Electronic Bandgap Materials, Ministry of Education, School of Physics and Electronic Engineering, Harbin Normal University, Harbin 150025, China

**Keywords:** NiMoO_4_, supercapacitor, nanofiber, electrospinning

## Abstract

One-dimensional NiMoO_4_ nanofibers were successfully prepared by electrospinning and high-temperature calcination. The supercapacitor performance tests were conducted on the prepared materials in a three-electrode system, and it was found that the calcination temperature during the preparation of the fibers seriously affects the final morphology and electrochemical performance of the obtained samples. The sample with a calcination temperature of 500 °C has better performance, its specific capacitance can reach 1947 F g^−1^, and the retention rate is 82.35% after 3000 cycles of constant current charging–discharging. The improvement of electrochemical performance is primarily on account of the unique one-dimensional nanostructure of the material, which can both enhance the charge transfer efficiency and effectively increase the speed of electrolyte ion diffusion.

## 1. Introduction

In recent years, due to the consumption of fossil fuels and environmental damage, people have now feared that using non-renewable fossil energy will bring about air pollution and a rise in average global temperatures. Meanwhile, new energy has become the most urgent need for sustainable development of a global nature in the 21st century [1,2]. Over the past decade, the drive to replace fossil fuels with biofuels, wind power, tidal power, and energy storage has become more urgent [3,4]. At present, supercapacitors are one of the more important energy storage devices [5,6,7]. Because of its advantages of fast charging–discharging capability, high flexibility [8,9], and long cycle life, it can provide storage technology to help solve the energy crisis.

Transition metal oxide is a kind of supercapacitor capacitive material that has been widely studied [10,11,12]. This kind of material can have different oxidation states and high theoretical capacitance when the Faraday redox reaction occurs. At present, bimetallic transition metal oxides, for instance, Nickel–cobaltate (NiCo_2_O_4_), Nickel–manganese (Ni-MnBMO), Calcium–molybdate (CaMoO_4_), Nickel–Tungstate(NiWO_4_) and Nickel–molybdate (NiMoO_4_) have become a research hotspot in the field of new energy storage materials because of their outstanding electrochemical properties and superior electrical conductivity [13,14,15,16,17,18].

Among various available transition metal oxides, NiMoO_4_ has attracted extensive attention due to its advantages such as good electrochemical conductivity, high theoretical capacitance value (2500 Fg^−1^), high cost effectiveness, rapid redox activity, low cost, and non-toxicity [19,20,21]. At present, spinel structure oxide nickel molybdate has been widely used in lithium batteries and supercapacitors. For NiMoO_4_ powder materials, the repeated ion embedding and extraction process will lead to volume expansion, and the loose connection between particles will also increase its own resistance, thus affecting its theoretical capacitance value cannot be maximized. How to further improve the capacitive properties of such materials is a meaningful research topic [22]. The above problems can be solved by constructing one-dimensional nanostructures to preserve the chemical properties of materials while ensuring the nanocrystallization of basic particles [23,24]. This kind of NiWO_4_ nanofiber is expected to present several advantages: First of all, the ultra-long ordinate axis with 1D nanostructure can provide more efficient transport pathways for both electrons as well as ions with greater electrochemical reversibility and cycle stability. Secondly, the crosslinked network nanofibers composed of individual NiWO_4_ nanoparticles can effectively decrease their surface energy. More importantly, the NiWO_4_ nanofibers would possess a larger interspace and a doubled active surface area, resulting in enhanced charge transport and electrochemical performance.

In this work, one-dimensional NiMoO_4_ nanofibers were prepared by simple electrospinning and calcination using nickel acetate and ammonium molybdate as reactants. The morphology and structure of nanofibers were controlled by a simple method of adjusting the calcination temperature. X-ray diffractometer (XRD), scanning electron microscope (SEM), transmission electron microscope (TEM), and X-ray photoelectron spectrometer (XPS) were used to characterize the morphology, structure, and chemical compositions of the prepared materials. The electrochemical properties of the materials were investigated through a three-electrode test system, and the corresponding structure–activity relationship was explained rationally.

## 2. Experiment

### 2.1. Chemicals

Polyacrylonitrile (PAN, Mw ~150,000) was purchased from Sigma-Aldrich Corporation (Shanghai, China). Ni(CH_3_COO)_2_, (NH_4_)_6_Mo_7_O_24_·4H_2_O, and N, N-dimethylformamide (DMF) were purchased from Zhiyuan Reagent (Tianjin, China). All chemicals were used directly after purchase without any further purification.

### 2.2. NiMoO_4_ Nanofibers’ Synthesis Method

In a typical synthesis process, dissolve 0.5 g polyacrylonitrile (PAN) powder in 5 mL N, n-dimethylformamide (DMF) and stir the above mixture for 1 h. After that, 0.174 g of Ni(CH_3_COO)_2_ and 0.124 g of (NH_4_)_6_Mo_7_O_24_·4H_2_O were added to the above solution and stirred at room temperature for 12 h. Next, a well-mixed precursor solution was injected into the syringe with a plastic needle, and the distance between the syringe needle and the aluminum foil collector was controlled at 12 cm. The operating voltage was set to 6 kV and electrospinning under dry conditions for several hours to obtain light green nanofibers. The samples of NiMoO_4_ nanofibers were synthesized by calcining the composite nanofibers at the temperatures of 450, 500, 600, and 700 °C and lasted for 2 h in air with a heating rate of 2 °C min^−1^. As-obtained NiMoO_4_ nanofibers were named NMO-450, NMO-500, NMO-600, and NMO-700 NFs, respectively.

### 2.3. Characterization

The structures of the prepared samples were collected by XRD (Rigaku D/max2600, Tokyo, Japan) which analyses with a Cu Kα radiation source (λ = 0.154178 nm), and the morphologies were characterized by SEM (SU70, Hitachi, Tokyo, Japan) and TEM (FEI, Tecnai TF20, Tokyo, Japan). The Brunauer Emmett Teller (BET) test was measured for the specific surface area of the samples. In addition, the chemical composition and element state of the samples were obtained from XPS.

### 2.4. Electrochemical Measurement Technology

The nature of the electrochemical property for as-prepared was investigated by a standard three-electrode system which was tested in 1 M KOH solution. Saturated calomel electrode (SCE) and Pt foil electrode with 1 cm^2^ were regarded as the reference electrode and counter electrode, respectively. The electrode slurry was prepared by evenly mixing the active substance, acetylene black and polytetrafluoroethylene (PTFE) binder in a mortar at a weight ratio of 8:1:1. Then, the mixed electrode slurry was equably coated on 1 cm^2^ nickel foam and dried under 60 °C vacuum for 12 h to obtain the working electrode. In this work, all the electrochemical data were tested by the electrochemical workstation (VMP3, France). The obtained data from the cyclic voltammetry (CV) test were collected by an optimal voltage range from 0 to 0.5 V at the scan rates for 5, 10, 20, 30, 40, and 50 mV s^−1^. Galvanostatic charging–discharging (GCD) processes were attained at the current densities of 1, 2, 4, 6, 8, and 10 A g^−1^ with the voltage range 0~0.44 V, respectively, according to the following equation:

(1) $$C_{m}=I\times ∆t/m\times ∆V$$

where *C_m_* represents the specific capacitance (F g^−1^), *I* is the discharge current (A), *m* is the mass of electrode material (mg), Δ*t* refers to the discharge time (s), and Δ*V* is the voltage drop upon discharging (V).

## 3. Results and Discussion

**Figure 1:**
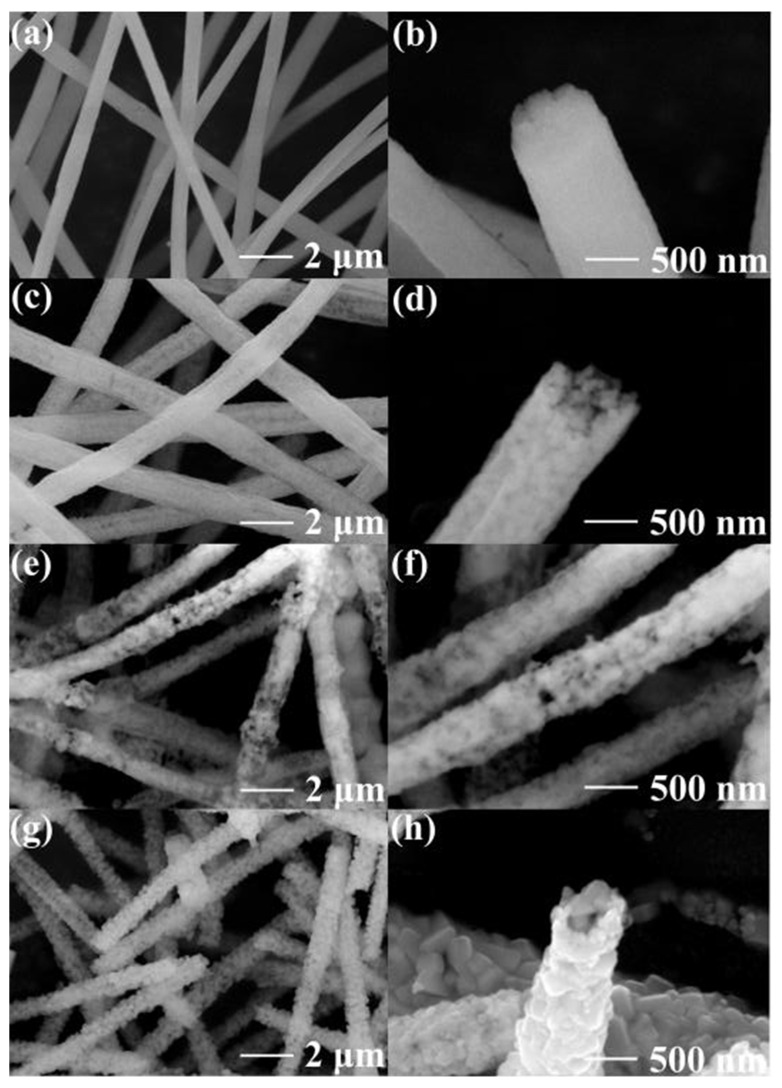
SEM images of (**a**) and (**b**) NMO-450, (**c**) and (**d**) NMO-500, (**e**) and (**f**) NMO-600, and (**g**) and (**h**) NMO-700 NFs under different magnifications.

The microstructure and morphological information were shown through SEM. Compared with the sample of NMO-450 (Figure 1a,b), NMO-600 (Figure 1e,f), and NMO-700 NFs (Figure 1g,h), the sample of NMO-500 NFs possess a well-organized 1D nanostructure as shown in Figure 1c,d. At 450 °C, the polymer template (PAN) served as a mighty carrier for the NiMoO_4_ structure, while at 500 °C, PAN powder was completely removed with air. Subsequently, at 600 °C, the pores between the nanoparticles grew larger. More interestingly, at 700 °C, nanofibers still preserved a 1D structure, although the partial samples appeared an issue of local fracture. These results show that it is feasible to obtain NiMoO_4_ with a special 1D structure through annealing the nanofibers at different high temperatures.

**Figure 2:**
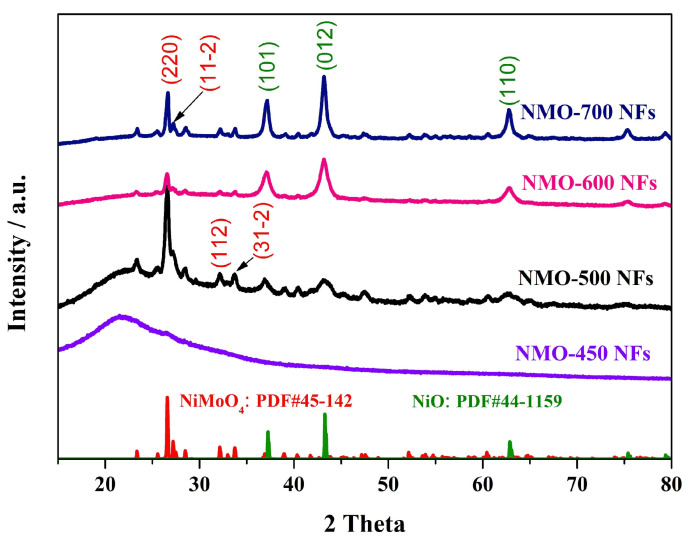
XRD patterns of NMO-450, NMO-500, NMO-600, and NMO-700 NFs.

The crystal structures of the NMO-450, NMO-500, NMO-600, and NMO-700 NFs were represented by XRD patterns (Figure 2). The characteristic peaks which located at 23.36, 26.58, 27.31, 32.3, 34.18, 36.68, 43.8, and 47.4° belong to (0 2 −1), (2 2 0), (1 1 −2), (4 0 0), (2 2 2), (4 2 −2), (2 4 −1), and (2 0 −4) lattice planes of NiMoO_4_ (JCPDS No. 45-0142), respectively. In the sample of NMO-450 NFs, the observed maxima are associated with the turbostratic structure typical of the char. Moreover, for the sample of NMO-600 and NMO-700 NFs, a few impurity peaks located at 37.03, 43.16, and 63.71° are assigned to (1 0 1), (0 1 2) and (1 1 0) lattice planes of NiO (JCPDS No. 44-1159). We believe that a pure phase NMO-500 NF with a one-dimensional nanostructure can be prepared under the appropriate temperature (500 °C) and the cooperative contribution of flexible electrospinning technology. These Mmonometallic oxides (NiO impurity) might be formed in a thermal oxidizing atmosphere at high temperatures. It can be seen from the XRD pattern that with the increase in calcination temperature, NiO becomes the main component of the sample. The electrochemical properties of the monometallic oxides are commonly lower than the bimetallic oxides, which hinder and impact the capacitive performances of pure NiMoO_4._

**Figure 3:**
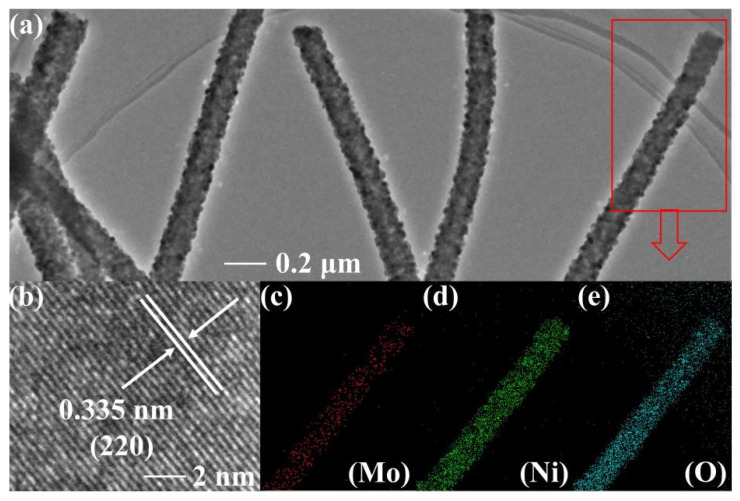
(**a**,**b**) TEM and HRTEM images of NMO-500 NFs, and (**c**–**e**) their EDX elemental mapping images.

**Figure 4:**
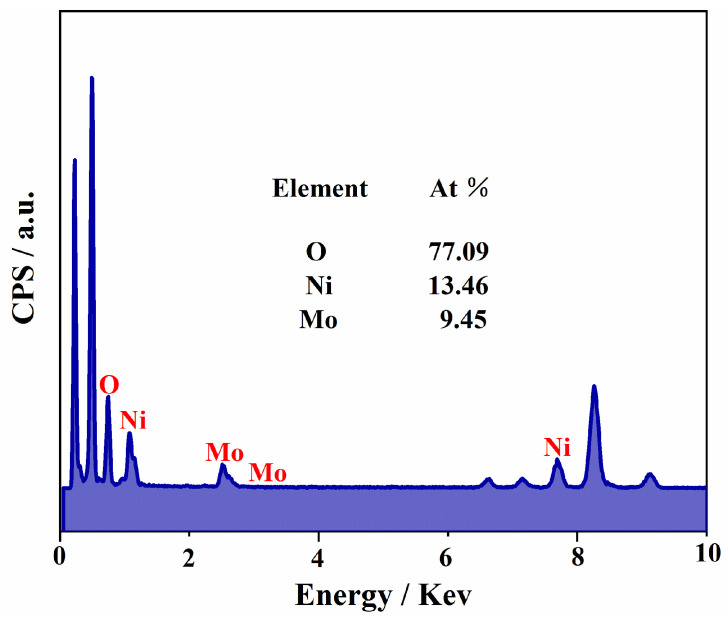
EDS of the NMO-500 NFs.

NMO-500 NFs’ morphologies and structures could be further characterized by TEM and HRTEM analysis. The NMO-500 NFs exhibit obvious one-dimensional nanostructures, which are coated with a layer of interconnected nanoparticles on the nanofibers (Figure 3a). As a result, the NMO-500 NFs have a relatively rough surface. The lattice spacing of the NMO-500 NFs was observed to be 0.335 nm, which corresponded to the (2 2 0) lattice plane of the spinel NiMoO_4_ (Figure 3b). Furthermore, the element surface distribution of Mo, Ni, and O elements for the as-fabricated samples significantly exhibit a high degree of overlap in the mapping (Figure 3c–e). This result strongly confirmed that the NiMoO_4_ nanofibers were successfully prepared. And on top of all the above, the element composition was also outlined through the energy element analysis data using an energy dispersive spectrometer (EDS) (Figure 4).

**Figure 5:**
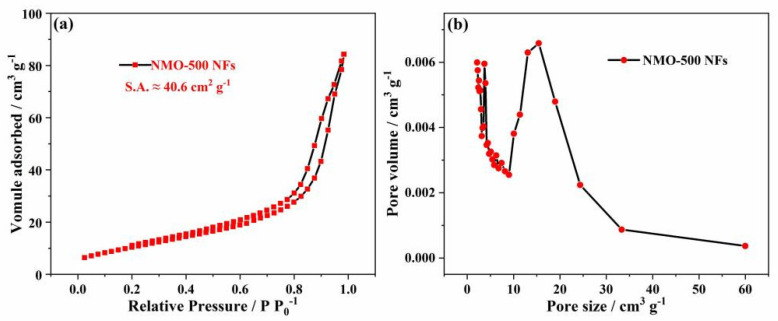
N_2_ adsorption–desorption isotherm loop for (**a**) NMO-500 NFs and (**b**) the curves of pore size distribution.

The BET-specific surface area and the pore size distribution of NMO-500 NFs were measured and analyzed via the N_2_ adsorption and desorption test. The NMO-500 NFs are type-IV isotherm curves (Figure 5a). NMO-500 NFs’ BET-specific surface area is apparently high (40.6 m^2^ g^−1^), which could perhaps be related to its one-dimensional structural characteristics and advantages. In addition, the pore size distribution of NMO-500 NFs has a pore distribution peak between 5 and 10 nm, indicating that the prepared sample is a mesoporous structure. (Figure 5b). This feasibly affords an advantageous path for the rapid diffusion of ions. Therefore, the NMO-500 NF, with its unique structural advantages, will provide pseudocapacitance to achieve the desired electrochemical characteristics of energy storage.

**Figure 6:**
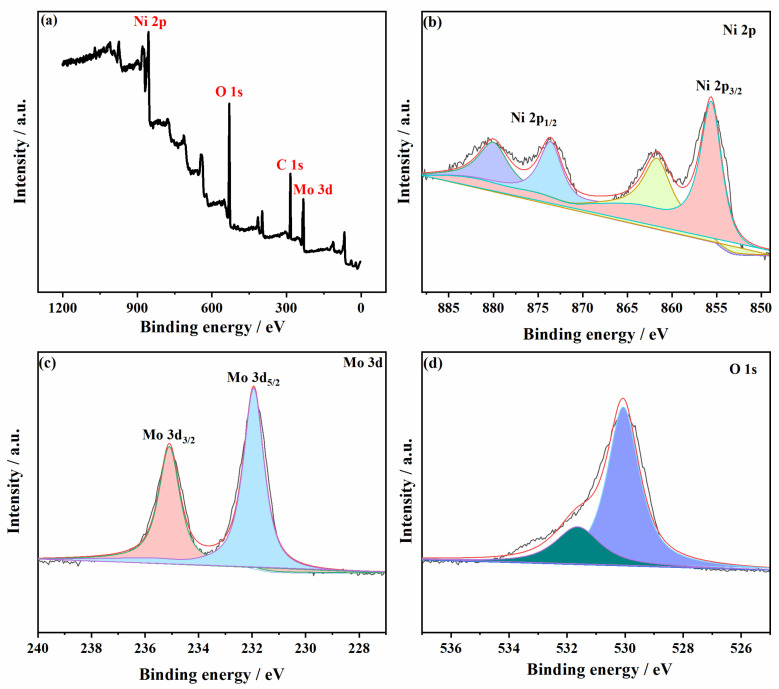
XPS characterization of NMO-500 NFs: (**a**) survey spectra, (**b**) nickel, (**c**) molybdenum, and (**d**) oxygen.

In order to determine the surface electronic states and chemical compositions of the NiMoO_4_ samples, XPS measurements were performed. XPS pattern spectra show the full spectrum of the NMO-500 NFs; there are four peaks located at 231.70, 284.00, 530.80, and 854.60 eV, matching with Mo 3d, C 1s, O 1s, and Ni 2p energy levels, respectively (Figure 6a). Among them, it could be deconvoluted as four peaks for Ni 2p core level spectra which peaks located at 855.90 and 861.50 eV (satellite peak) correspond to Ni 2p_3/2_ energy level, whereas the peaks located at 873.7 and 880 eV (satellite peak) ascribe to the Ni 2p_1/2_ energy level. As shown in Figure 6b, there is a binding energy gap of 17.80 eV between the main peaks of Ni 2p_3/2_ and Ni 2p_1/2_, which indicates that Ni^2+^ is an oxidation state. The Mo 3d energy level spectra show two peaks located at 232.00 and 235.10 eV which correspond to Mo 3d_5/2_ and Mo 3d_3/2_ (Figure 6c). And there is a binding energy gap of 3.10 eV for separated Mo 3d, which represents the oxidation state of Mo^6+^. The peak of binding energy which is located at 530.79 eV belongs to the O 1s energy level of NiMoO_4_ and the peak at 531.40 eV implies the generation of low coordination oxygen ions on the surface of the sample (Figure 6d). Thus, the XPS analysis results revealed the generation of the nanostructure NiMoO_4_, and confirmed the formation of NiMoO_4_ nanoparticles embedded in nanofibers, as already analyzed by the XRD pattern.

**Figure 7:**
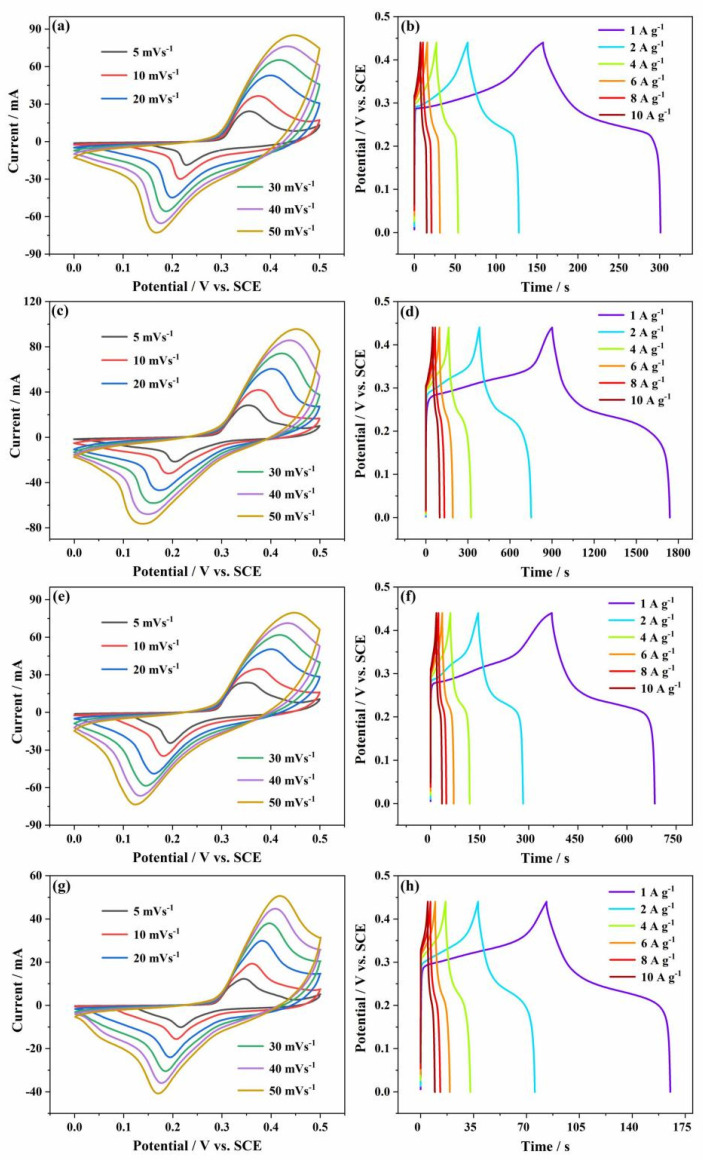
CV curves of (**a**) NMO-450 NFs, (**c**) NMO500 NFs (**e**) NMO-600 NFs, and (**g**) NMO-700 NFs at different scan rates in 1 M KOH electrolyte; GCD curves of (**b**) NMO-450 NFs, (**d**) NMO-500 NFs, (**f**) NMO-600 NFs, and (**h**) NMO-700 NFs at different current densities in 1 M KOH electrolyte.

The CV curves of NMO-450 NFs, NMO-500 NFs, NMO-600 NFs, and NMO-700 NFs at the scan rates from 5 to 50 mV s^−1^ were shown in Figure 7a,c,e,g. The CV curves of NMO-500 NFs possessed symmetrical and stable redox current peaks with a small potential drift and good repeatability when the scan rate increased, and their integrated area without apparent deformation, suggesting that the NMO-500 NFs have a good electrochemical reversibility derived from the improved nanostructures for adapting the desired fast charge and discharge reactions.

Figure 7b,d,f,h show the GCD curves of NMO-450 NFs, NMO-500 NFs, NMO-600 NFs, and NMO-700 NFs at different current densities of 1, 2, 4, 6, 8, and 10 A g^−1^ with the voltage range from 0 to 0.44 V. Distinctly, with the gradual increase in the current density, the charging–discharging time was significantly reduced, which is because the ions could more fully diffuse deep into the electrode material at the lower current density and promote the specific capacitance. In addition, the slope of all GCD curves changes nonlinearly with the increase in charging–discharging time, which further elucidates the representative pseudo-capacitance performance generated by the electrochemical redox reaction of NMO-500 NFs at the electrode–electrolyte interface.

**Figure 8:**
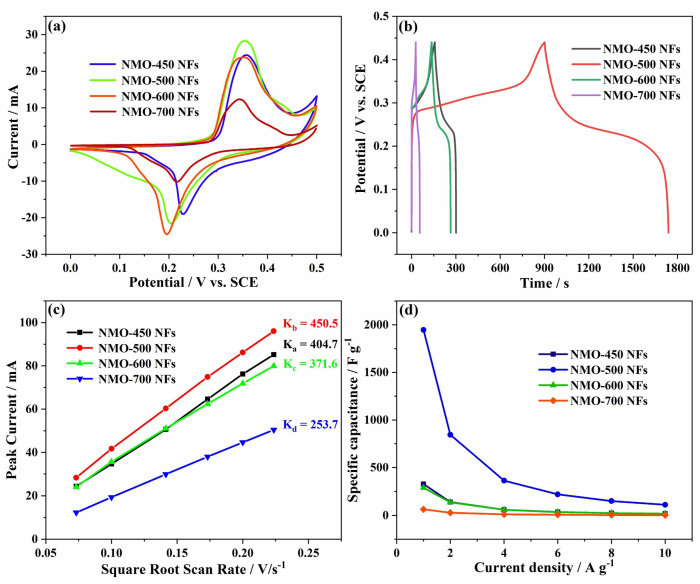
(**a**) CV curves of NMO-450 NFs, NMO-500 NFs, NMO-600 NFs, and NMO-700 NFs at 5 mV s^−1^ in 1 M KOH electrolyte. (**b**) GCD curves of NMO-450 NFs, NMO-500 NFs, NMO-600 NFs, and NMO-700 NFs at 1 A g^−1^ in 1 M KOH electrolyte. (**c**) Linear relationship of peak current vs. square root of scan rates of the NMO-450 NFs, NMO-500 NFs, NMO-600 NFs, and NMO-700 NFs electrode materials. (**d**) Mass-specific capacitances of pristine NMO-450 NFs, NMO-500 NFs, NMO-600 NFs, and NMO-700 NFs at different current densities.

As demonstrated in Figure 8a,b, the prepared samples’ electrochemical performances with well-designed nanostructures were estimated through combining the measured results of CV and GCD curves. The representative CV curves of NMO-450 NFs, NMO-500 NFs, NMO-600 NFs, and NMO-700 NFs which were supported at the scan rate of 5 mV s^−1^ were presented in Figure 8a. A pair of obviously and approximately symmetric sharp redox peaks in every CV curve were present, which indicated the role of the Faraday reaction (Ni^2+^/Ni^3+^) in charge storage. In addition, we found that the Mo element did not participate in the entire reaction and was still present in the sample for the form of polycation MoO_4_^2−^; meanwhile, there was no redox peak of Mo in the CV curve, which also confirmed the same results. It could only increase the conductivity of Nickel molybdate, thus improving the supernal specificity. The CV curve showed that the redox peaks remained symmetrical and also retained their shape, even at higher scanning rates, which indicated the reversibility of the reaction and the glorious rate capability of the electrode material. It was significantly more outstanding than those of NMO-450 NFs, NMO-600 NFs, and NMO-700 NFs for the electrochemical response current density and CV curve of NMO-500 NFs, and combined with XRD pattern analysis, which may be due to the presence of impurities (NiO) in the sample. In addition, the charging–discharging curves of a series of samples when the current density is 1 A g^−1^ were shown in Figure 8b. The curves of NMO-450, NMO-500, NMO-600, and NMO-700 had representative asymmetry and good reversibility, showing Faraday pseudocapacitance behavior. The discrepant specific capacitance for different electrode materials can be calculated by Equation (1). Notably, at a current density of 1 A g^−1^, the NMO-500 HNFs were more efficient such as showing a longer discharge time and a higher 1947 F g^−1^ specific capacitance than the NMO-450 NFs (328 F g^−1^), NMO-600 NFs (290 F g^−1^), and NMO-700 NFs (63 F g^−1^). These results were corresponded with the analysis of CV curves. It is well known that in reversible or quasi-reversible reactions, the peak current is proportional to the square root of the scanning rate (*ν*^1/2^). And, the first-order linear constant *K* of the ionic diffusion rate can effectively reflect the diffusion and transport rate of ionic electrolyte during the electrochemical reaction. NMO-500 NFs (*K*_b_~450.5) presented a higher value of *K* than that of NMO-450 NFs (*K*_a_~404.7), NMO-600 NFs (*K*_c_~371.6), and NMO-700 NFs (*K*_d_~253.7), which suggested that the one-dimensional nanostructure has more advantages than bulk nanostructure, particularly during the ion transport process (Figure 8c). More significantly, NMO-500 NFs manifested an apparent pseudocapacitive property, which may include the diffusion-controlled insertion behavior and surface capacitive effects. Furthermore, according to the GCD curves in Figure 8d, the variation law of specific capacitance can be calculated accurately. When the current density increases from 1 to 10 A g^−1^, the specific capacitance of the NMO-450 NFs, NMO-500 NFs, NMO-600 NFs, and NMO-700 NFs decreases significantly. This phenomenon is possible given the limitation of electrolyte diffusion in conjunction and contact resistance with the electrochemical reaction process, resulting in a decrease in capacitance value as the current density increases. Meanwhile, this demonstrates that NMO-500 NFs have a higher specific capacitance and significant rate capability than most pure NiMoO_4_ or NiMoO_4_ matrix composites through different experimental parameters, as shown in Table 1.

**Figure 9:**
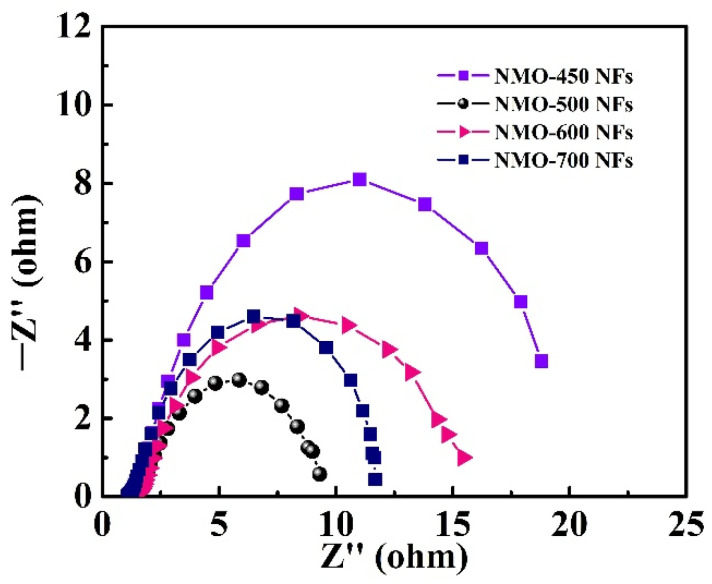
Nyquist plots of the NMO-450 NFs, NMO-500 NFs, NMO-600 NFs, and NMO-700 NFs.

Furthermore, the Faraday reaction on the electrode surface was evaluated by EIS. The Nyquist plots of NMO-450 NFs, NMO-500 NFs, NMO-600 NFs, and NMO-700 show the standard shape in the high-frequency zone (Figure 9). In addition, the equivalent series resistance (Rs) can be calculated by interception of the Z′-axis, and the diameter distance in the impedance spectrum matches the transfer charge resistance (Rct). It is clearly shown that the electrochemical impedance spectroscopy of the NMO-500 NFs showed the lowest values of Rs and Rct among these catalysts.

**Figure 10:**
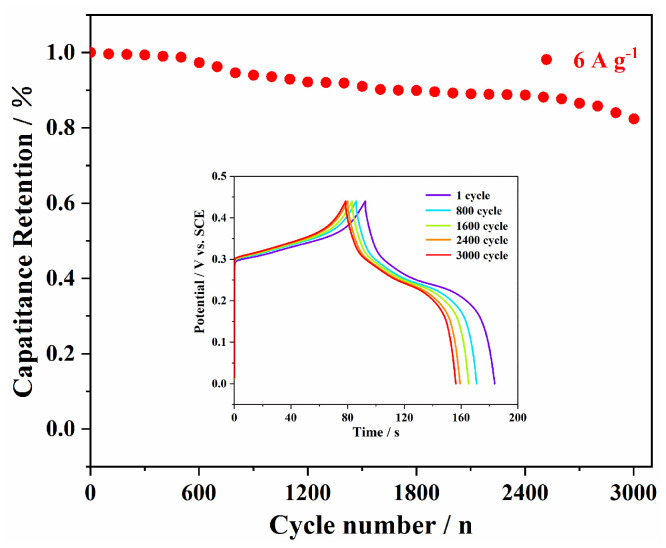
Cycling stability of NMO-500 NFs at a current density of 6 A g^−1^ for 3000 cycles, and (inset) the galvanostatic charge/discharge curves of the NMO-500 NFs over the 1st, 800th, 1600th, 2400th and 3000th cycles at a current density of 6 A g^−1^.

For evaluating the cycling properties of the electrodes, the values of capacitance retention were calculated by charging–discharging tests at the current density of 6 A g^−1^ (Figure 10). The NMO-500 NFs still maintained an excellent capacitance retention rate (~82.35%) over 3000 cycles at high current density (6 A g^−1^). In addition, as shown in the inset of Figure 9, the typical GCD curves of NMO-500 NFs still revealed a linear correlation of potential–time after the 1st, 800th, 1600th, 2400th, and 3000th circles, respectively. These results indicated the NMO-500 NFs with unique 1D nanostructures show excellent long-term electrochemical stability and characteristics of ideal capacitors. Therefore, it may be due to its distinct nanostructure that leads to improved cyclic stability, which not only increases the contact area of electrons and ions but also shortens the path between the electrolyte and active materials.

## 4. Conclusions

In this work, one-dimensional nanostructured molybdenum nickel oxide (NiMoO_4_) electrode with excellent electrochemical properties was constructed at an optimal temperature (500 °C) using an innovative electrospinning technique. NiMoO_4_ nanofibers exhibit excellent specific capacitance (1947 F g^−1^) and outstanding capacitance retention (82.35%) undergoing the 3000 cycles under 6 A g^−1^ conditions. This result can be attributed to their unique 1D nanostructure, which eliminated the negative effect of the powder agglomerates and exhibited good crystallinity at an appropriate temperature. This shows that it can be widely used in various portable electronic devices and provides a reference for the preparation of NiMoO_4_-based electrode materials with excellent electrochemical properties and good cycle stability.

## Figures and Tables

**Table 1:** Specific capacitance and cycle life of the NMO-500 NFs electrode material for comparison with as-reported related electrodes.

Electrode Material	Electrolyte	Specific Capacitance	Cycle Life	Ref.
NiMoO_4_ nanospheres	3 M KOH	974.4 F g^−1^ (at 1 A g^−1^)	75% (2000th at 5 A g^−1^)	[14]
NiMoO_4_	2 M NaOH	392.53 F g^−1^	87.14% (1000th at 5 A g^−1^)	[17]
NiMoO_4_ nanorods	3 M KOH	672 F g^−1^ (at 4 A g^−1^)	72% (1000th at 1 A g^−1^)	[25]
NiMoO_4_	6 M KOH	594 F g^−1^ (at 1 A g^−1^)	56% (1000th at 1 A g^−1^)	[26]
NiMoO_4_/MWCNTs	3 M KOH	805 F g^−1^ (at 1 A g^−1^)	66.7% (1000th at 1 A g^−1^)	[27]
NiMoO_4_/CoMoO_4_ nanorods	1 M KOH	1445 F g^−1^ (at 1 A g^−1^)	78.8% (3000th at 10 A g^−1^)	[28]
NiMoO_4_/3D-rGO (II)	3 M KOH	932 F g^−1^ (at 1 A g^−1^)	76% (500th at 1 A g^−1^)	[29]
MnO_2_/NiMoO_4_ nanostructure	5 M KOH	918 F g^−1^ (at 1 A g^−1^)	80% (10000th at 5 A g^−1^)	[30]
NiCo_2_O_4_@NiWO_4_ core–shell nanowire	6 M KOH	1384 F g^−1^ (at 1 A g^−1^)	87.6% (6000th at 5 A g^−1^)	[31]
Amorphous NiWO_4_	2 M KOH	586.2 F g^−1^ (at 0.5 A g^−1^)	91.4% (5000th at 2A g^−1^)	[32]
NiMoO_4_ NFs	1 M KOH	1947 F g^−1^ (at 1 A g^−1^)	82.35% (3000th at 6 A g^−1^)	this work

## Data Availability

All the relevant data are included in this published article.
